# Analysis of Synergism between Extracellular Polysaccharide from *Bacillus thuringensis* subsp. *kurstaki* HD270 and Insecticidal Proteins

**DOI:** 10.3390/toxins15100590

**Published:** 2023-09-28

**Authors:** Bai Xue, Meiling Wang, Zeyu Wang, Changlong Shu, Lili Geng, Jie Zhang

**Affiliations:** 1 College of Life Sciences, Northeast Agricultural University, Harbin 150030, China; 2 State Key Laboratory for Biology of Plant Diseases and Insect Pests, Institute of Plant Protection, Chinese Academy of Agricultural Sciences, Beijing 100193, China

**Keywords:** *Bacillus thuringiensis*, microbial pesticides, extracellular polysaccharides, synergistic effects, BBMVs, midgut juice

## Abstract

*Bacillus thuringiensis* (Bt) is the most widely used biopesticide worldwide and can produce several insecticidal crystal proteins and vegetative insecticidal proteins (Vips) at different growth stages. In our previous study, extracellular polysaccharides (EPSs) of Bt strain HD270 were found to enhance the insecticidal activity of Cry1Ac protoxin against *Plutella xylostella* (L.) and promote the binding of Cry1Ac to the intestinal brush border membrane vesicles (BBMVs). Whether the synergistic activity of Bt EPSs is common to other Cry1-type or Vip proteins is unclear, as is the potential synergistic mechanism. In this study, crude EPS-HD270 was found to increase the toxicity of Cry1-type toxins and Vip3Aa11 against different lepidopteran pests by approximately 2-fold. The purified EPS-HD270 also possessed synergistic activity against the toxicity of Cry1Ac and Vip3Aa11 against *Spodoptera frugiperda* (J.E. Smith) and *Helicoverpa armigera* (Hübner). Furthermore, we found that EPS-HD270 had a strong binding ability with Vip3Aa11 and promoted the binding of Vip3Aa11 to the BBMVs of *H. armigera* and *S. frugiperda*. Bt EPS-HD270 also protected Vip3Aa11 from proteolytic processing in larval midgut juice. Bt EPSs had universal synergistic effects on Cry1-type or Vip toxins against *S. frugiperda* and *H. armigera*. Bt EPS-HD270 exhibited synergistic activity with Vip3Aa through promotion of binding to BBMVs and protection from digestion by midgut protease. The results indicated that synergistic activity with Bt toxins was an important function of Bt EPSs, which was very different from other *Bacillus* spp.

## 1. Introduction

Bacterial extracellular polysaccharides (EPSs) are synthesized inside the cells and secreted into the extracellular environment or synthesized outside the cells by cell wall-anchored enzymes during bacterial growth and metabolism [1,2]. Exopolysaccharides are macromolecules composed of monosaccharide units that form complex structures and exhibit multiple functions. Bacteria produce extracellular polysaccharides to protect themselves from various environmental stresses, including desiccation, ionic stress, and other biotic stresses, such as predation by amoebae [3], facilitating their adaptation in different habitats by forming biofilms. *Bacillus* bacteria have also been reported to produce extracellular polysaccharides with flocculation activity [4], and the abilities to absorb lead [5] and induce systemic resistance to plant pathogenic bacteria [6], in addition to their well-known function in the biofilm matrix of *Bacillus subtilis* [7]. In our recent study, we found that most *Bacillus thuringiensis* (Bt) strains could produce extracellular polysaccharides. EPS produced by the Bt HD270 strain increased the activity of insecticidal crystal proteins [8].

Bt is an aerobic, Gram-positive entomopathogenic bacterium [9] that produces insecticidal proteins at different growth periods [10]. These proteins are commonly known as crystal (Cry) proteins when produced during the spore-forming stage and vegetative insecticidal proteins (Vips) when produced at the vegetative growth stage. Due to their specific insecticidal activity against a large number of lepidopteran and coleopteran pests in agriculture, they are widely used in biopesticides and transgenic crops [11]. The two types of proteins have different structures and different insecticidal mechanisms, but both need to bind to receptors in the intestinal brush border membrane vesicles (BBMVs) to exert insecticidal activity.

The exploration of Bt strains and insecticidal proteins has entered a bottleneck period, making it difficult to explore new insecticidal resources. Therefore, there is growing interest in synergistic substances that could improve the larvicidal activity of Bt proteins. In our previous study, extracellular polysaccharides produced by the Bt strain HD270 (EPS-HD270) were observed to increase the toxicity of the total toxins isolated from HD270 and Cry1Ac proteins against *Plutella xylostella* (L.) [8]. The molecular weight of EPS-HD270 was 58.0 kDa, and EPS-HD270 was composed mainly of mannose (44.2%) and GlcN (35.5%) [8]. However, it is not clear whether Bt EPSs would commonly increase the toxicity of both Cry and Vip proteins against different target insect pests. Clarification of this issue will reveal multi-biological functions of Bt EPSs and lay a theoretical foundation for using EPSs to improve the control effect of Bt formulations.

In this study, the synergistic effects of EPS with Cry and Vip proteins were analyzed. EPS-HD270 not only enhanced the toxicity of the Cry1Ac, Cry1Ab, and Cry1Ah proteins but also increased that of the Vip3Aa11 protein against *Helicoverpa armigera* (Hübner) and *Spodoptera frugiperda* (J.E. Smith). The potential mechanism involved in the synergism of EPSs and Vip3Aa11 toxin was the protection of Vip3Aa11 from proteolytic processing in larval midgut juice and the increased binding affinity of Vip3Aa11 to BBMVs of *H. armigera* and *S. frugiperda* caused by adding EPSs.

## 2. Results

### 2.1. Universal Synergistic Activity of Bt EPSs on Insecticidal Proteins

**Figure 1:**
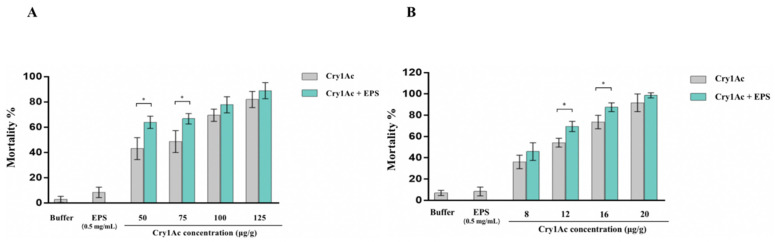
Insecticidal activity of Cry1Ac protein by adding purified EPS of Bt HD270 against *S. frugiperda* (**A**) and *H. armigera* (**B**). Data are the average ± standard deviation (SD) from three independent experiments. (* *p* < 0.05).

EPS-HD270 was found to enhance the virulence of the Cry1Ac protoxin against *P. xylostella* in our previous work [8], but the universality of the synergistic effect is unclear. In this study, the synergistic activities of EPS-HD270 with Cry1Ac protein against larvae of other lepidopteran insect pests, including *S. frugiperda* and *H. armigera*, were further analyzed. The LC_50_ values of Cry1Ac protoxin against first instar larvae of *S. frugiperda* and *H. armigera* were 68.26 μg/g and 12.40 μg/g, respectively, while the LC_50_ values of Cry1Ac protoxin supplemented with 2.0 mg/g crude EPS were 33.64 μg/g and 5.92 μg/g (Table 1), respectively, indicating a 2.03-fold and 2.09-fold increase in insecticidal activity. Crude EPS was treated with proteinase K and further purified with an anion-exchange chromatography column and a gel filtration chromatography column, as in our previous study [8], to obtain a single component (shown in Figure S1). Moreover, the addition of 0.5 mg/g purified EPS significantly increased the 7-day mortality of 50 and 75 μg/g Cry1Ac protoxin against *S. frugiperda* to 63.9% and 66.7% (*p* < 0.05, Figure 1A); 7-day mortality of 12 and 16 μg/g Cry1Ac protoxin against *H. armigera* increased to 69.4% and 87.5% (*p* < 0.05, Figure 1B). However, crude and purified EPS showed no toxicity against *S. frugiperda* and *H. armigera* larvae.

**Figure 2:**
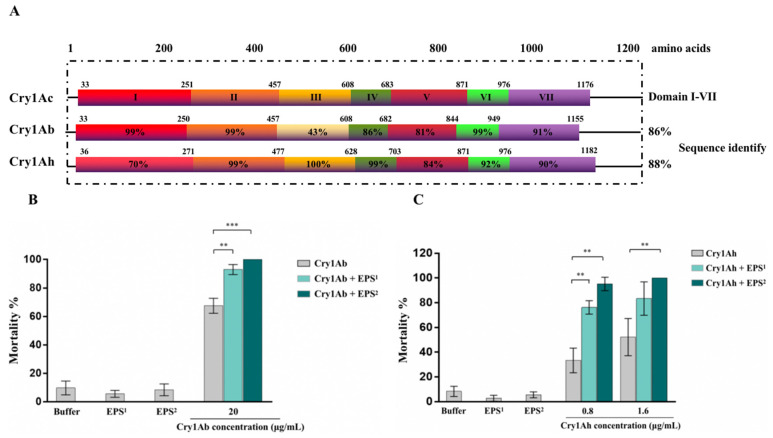
Insecticidal activity of crude EPS−HD270 and Cry1A protein against *P. xylostella*. (**A**) Analysis of the amino acid sequence identity of Cry1Ab and Cry1Ah with Cry1Ac protoxins. Percentage represents amino acid sequence identity with domain I to VII of Cry1Ac protein. (**B**) Cry1Ab protein and EPS. ^1^ means the concentration of crude EPS was 5 mg/mL. ^2^ means the concentration of crude EPS was 50 mg/mL. (**C**) Cry1Ah protein and EPS. Data are the mean value ± standard deviation from three independent experiments. (** *p* < 0.01, *** *p* < 0.001).

In addition, the synergistic activity of EPS-HD270 with Cry1Ab (which shares 86% of the amino acid sequence identity of Cry1Ac) and Cry1Ah protein (which shares 88% of the amino acid sequence identity of Cry1Ac) was investigated (Figure 2A). The bioassay results showed that the 3-day mortality of 20 μg/mL Cry1Ab against *P. xylostella* was increased to 92.9% (*p* < 0.01) and 100.0% (*p* < 0.001) by adding 5 and 50 mg/mL crude EPS, respectively (Figure 2B). The 3-day mortality of 0.8 μg/mL Cry1Ah against *P. xylostella* was increased to 76.2% (*p* < 0.01) and 95.2% (*p* < 0.001) by adding 5 and 50 mg/mL crude EPS, respectively, and the mortality of 1.6 μg/mL Cry1Ah was also significantly increased to 100.0% (*p* < 0.01) by adding 50 mg/mL crude EPS (Figure 2C). Overall, Bt EPS-HD270 enhanced the virulence of the Cry1Ac protoxin against lepidopteran insect pests, including *P. xylostella*, *S. frugiperda* and *H. armigera*, and synergistic activities of EPS-HD270 with other Cry1-type proteins, including Cry1Ab and Cry1Ah, were observed.

**Figure 3:**
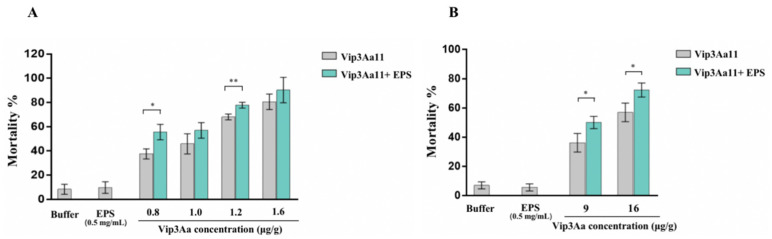
Insecticidal activity of Vip3Aa protein and purified EPS-HD270 EPS against *S. frugiperda* (**A**) and *H. armigera* (**B**). Data are the mean value ± standard deviation from three independent experiments. (* *p* < 0.05, ** *p* < 0.01).

In addition to Cry-1 type proteins with a typical three-domain structure, the effects of Bt EPSs on the toxicity of Vip3Aa were analyzed. The LC_50_ of Vip3Aa protein against first instar larvae of *S. frugiperda* and *H. armigera* were 1.22 μg/g and 12.57 μg/g, respectively, while the LC_50_ of Vip3Aa protein added with 2.0 mg/g crude EPS were 0.49 μg/g and 5.51 μg/g (Table 2), indicating that the virulence was enhanced 2.49-fold and 2.28-fold, respectively. Furthermore, the addition of 0.5 mg/g purified EPSs significantly increased the 7-day mortality of 0.8 and 1.2 μg/g Vip3Aa protein against *S. frugiperda* to 55.6% (*p* < 0.05) and 77.7% (*p* < 0.01, Figure 3A). The 7-day mortality of 9 and 16 μg/g Vip3Aa protein against *H. armigera* was also enhanced to 50.0% and 72.2%, respectively (*p* < 0.05, Figure 3B). In summation, Bt EPS-HD270 exhibited synergistic activities with Vip3Aa against *S. frugiperda* and *H. armigera* as well.

### 2.2. EPS-HD270 Formed Specific Binding with Vip3Aa11 Protein

**Figure 4:**
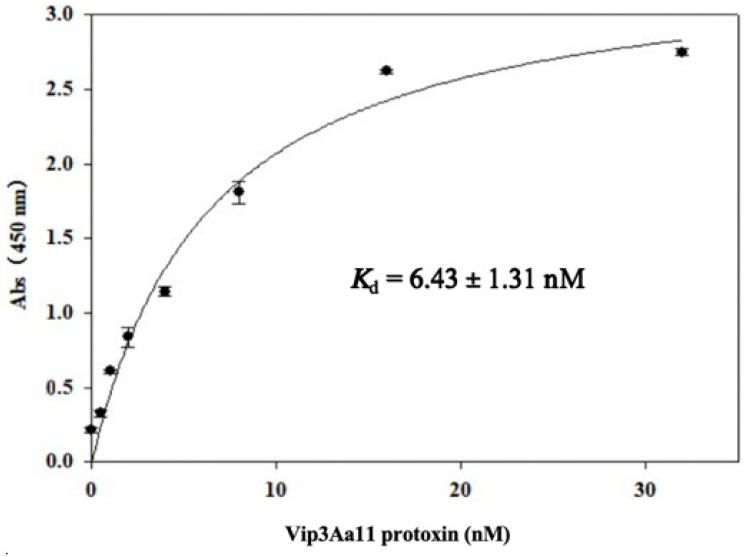
Analysis of the interaction between Vip3Aa11 and purified EPS. Error bars represent SD. Abs represents absorbance.

The Vip3A protoxin contains five distinct structural domains (domains I–V), and the carbohydrate-binding module domains were found in domains IV and V, which were exposed to the solvent [12,13]. We hypothesized that EPS might bind to the Vip3A protoxin, which in turn affects the toxicity of the Vip3A protein against *S. frugiperda* and *H. armigera*. The interaction between EPS-HD270 and the Vip3Aa protein was determined by ELISA using an anti-Vip3A antibody. The results showed that EPS-HD270 specifically bound to the Vip3Aa11 protoxin with a *K*_d_ value of 6.43 ± 1.31 nM (Figure 4).

### 2.3. EPS-HD270 Delayed Proteolytic Processing of Cry1Ac and Vip3Aa Proteins in Midgut Juice

**Figure 5:**
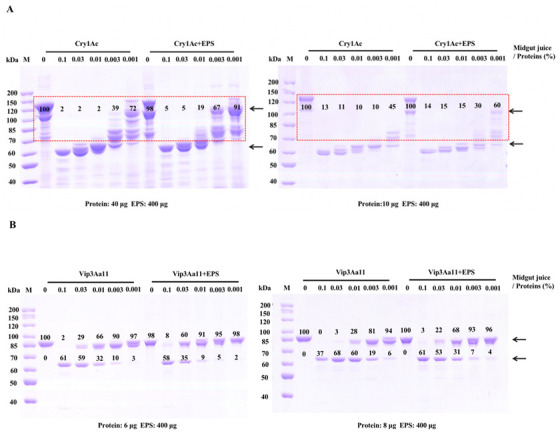
Proteolytic processing of Cry1Ac and Vip3Aa11 in midgut juice. (**A**) SDS-PAGE analysis of Cry1Ac digested by S. frugiperda (left) and H. armigera (right) midgut juice. The arrows indicate Cry1Ac protoxin (~130 kDa) and activated toxin (~65 kDa). (**B**) SDS-PAGE analysis of Vip3Aa11 digested by S. frugiperda (left) and H. armigera (right) midgut juice. The arrow indicates Vip3Aa11 protoxin (~88 kDa) and activated toxin (~66 kDa). M, molecular weight marker.

Purified EPS-HD270 specifically bound with Cry1Ac [8] and Vip3Aa, which might protect proteins from being digested by proteases in the midgut. The effects of EPS on the proteolytic processing of Bt proteins in midgut juice were analyzed. Cry1Ac and Vip3Aa proteins were mixed with purified EPS-HD270 in the proportions used in the bioassay. The intensity of the protein bands was determined using ImageJ software, and untreated Cry1Ac or Vip3Aa was used as a 100% reference. After 40 μg of Cry1Ac processing by *S. frugiperda* midgut juice ratios at 0.003% and 0.001%, the density of protein with a molecular mass higher than 70 kDa was approximately 39% and 72%, while with the addition of 400 μg EPS-HD270, the density of protein greater than 70 kDa was approximately 67% and 91% (Figure 5A). The same phenomenon was observed when 10 μg of Cry1Ac was mixed with 400 μg of EPS-HD270 processed with *H. armigera* midgut juice at ratios of 0.003% and 0.001% (Figure 5A). EPS-HD270 delayed proteolytic processing of Cry1Ac protoxin in the midgut juice of *S. frugiperda* and *H. armigera*.

After 6 μg of Vip3Aa protoxin processing with *S. frugiperda* midgut juice ratios at 0.03% and 0.01%, the density of protoxin was approximately 29% and 66%, while with the addition of 400 μg EPS-HD270, the density of protoxin was increased to 60% and 91%, respectively (Figure 5B). Similar results were obtained when the density of protoxin was increased to 68% and 93% with the addition of 400 μg of EPS-HD270 after processing with *H. armigera* midgut juice ratios at 0.01% and 0.003% (Figure 5B). Overall, EPS-HD270 delayed proteolytic processing of the Cry1Ac and Vip3A protoxins in the midgut juice of *H. armigera* and *S. frugiperda*.

### 2.4. EPS-HD270 Enhances the Binding Affinity of Vip3Aa11 to BBMVs of H. armigera and S. frugiperda

**Figure 6:**
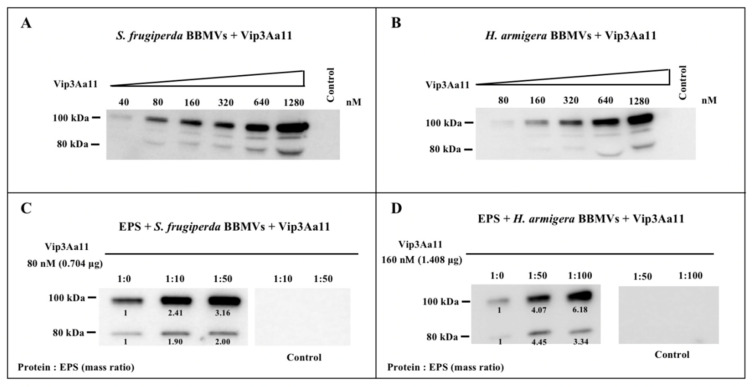
Binding assay of Vip3Aa11 protoxins and purified EPS to BBMVs of *S. frugiperda* and *H. armigera*. Saturation binding assays of Vip3Aa11 to *S. frugiperda* (**A**) and *H. armigera* BBMVs (**B**). The control represents the highest concentration of Vip3Aa11 without BBMVs, indicating that the toxins did not precipitate in the absence of BBMVs. (**C**) The effects of EPS on the binding ability of Vip3Aa11 (80 nM) to *S. frugiperda* BBMVs. (**D**) The effects of EPS on the binding ability of Vip3Aa11 (160 nM) to *H. armigera* BBMVs. Control represents the absence of BBMVs, and the result indicates that toxins mixed with EPS did not precipitate in the absence of BBMVs.

Cry1-type proteins and Vip3A proteins are pore-forming toxins, and the ability to bind to receptors on BBMVs is a primary factor affecting their virulence [14]. Our previous work found that EPS-HD270 promotes the binding ability of Cry1Ac protoxin to BBMVs of *P. xylostella*. Therefore, the effects of EPS on the binding of Vip3Aa11 protoxin to the BBMVs of *S. frugiperda* and *H. armigera* were further analyzed in the present study. Saturation binding assays were conducted by using increasing concentrations of Vip3Aa proteins to bind to the BBMVs of *H. armigera* and *S. frugiperda*. Western blot results showed that binding of Vip3Aa11 protein to BBMVs of these two insects was saturable (Figure 6A,B). Subsequently, 80 nmol/L of Vip3Aa11 at unsaturated binding was used, and the addition of EPS-HD270 at mass ratio of 1:10 and 1:50 enhanced the signal of Vip3Aa11 protoxin binding to BBMVs of *S. frugiperda* 2.41- and 3.16-fold (Figure 6C). In addition, for BBMVs of *H. armigera*, 160 nmol/L of Vip3Aa11 at unsaturated binding was used, and the addition of EPS-HD270 at mass ratio of 1:50 and 1:100 enhanced the signal of Vip3Aa11 protoxin binding to BBMVs 4.07- and 6.18-fold (Figure 6D). Thus, the EPS-HD270 interacted with Vip3Aa11 protoxin directly to enhance binding to BBMVs, which was correlated with increased toxicity against *S. frugiperda* and *H. armigera*.

## 3. Discussion

Extracellular polysaccharides of Bt exist in fermentation broth during production and are discarded as byproducts. In addition, due to the requirements of environmental protection, fermentation wastewater can only be discharged after strict treatment, which undoubtedly increases the production costs of Bt products, increases the economic burden on manufacturers, and increases costs for farmers using biological pesticides.

In this study, we found that exopolysaccharides produced by Bt increased the toxicity of Cry1-type and Vip3 proteins against Lepidopteran insect pests. In other words, EPS produced by excellent strains such as HD270 can improve the insecticidal activity of a variety of insecticidal proteins against a variety of insect pests. If these polysaccharides are effectively recycled and added to Bt products, it will reduce the environmental pollution caused by emissions; more importantly, it will improve the insecticidal effect of Bt products, save production costs, and be an innovative move that kills two birds with one stone. To our knowledge, Bt EPS were the first to exhibit synergistic activities with self-producing active substances of bacteria. This study lays a theoretical foundation for the production and application of Bt extracellular polysaccharides and provides important references for the creation of other biological pesticides.

Chakroun et al. reported that trypsin-activated Vip3Aa protein showed higher toxicity than nickel-purified Vip3Aa protoxin against *S. frugiperda* [15]. In this study, only nickel-purified Vip3Aa11 protoxin was used. Further study is needed to investigate the effects of EPS-HD270 on the activated Vip3Aa protein. In addition, some Bt toxins were found to evolve resistance to lepidopteran insect pests, including *P. xylostella* with resistance against Cry1Ac [16,17], *H. armigera* with resistance against Vip3Aa [18], and *S. frugiperda* with resistance against Cry1F and Cry1Ab [19,20,21]. Whether EPS produced by Bt can help Bt toxins deal with the resistance is a question that needs to be analyzed.

The synthesis of extracellular polysaccharides is an energy-consuming process [22]. Our previous work found that 96.5% of 170 Bt strains cultured in LB medium produced exopolysaccharides [8]. Exopolysaccharides produced by *Bacillus* can not only help *Bacillus* spp. deal with external pressures [7], but also improve their virulence to the hosts, which is very important for the infection and epidemic of Bt strains [8]. These important ecological functions may be the reasons why most Bt strains need to synthesize extracellular polysaccharides at the expense of energy.

Whether Cry1-type or Vip3 proteins are activated by proteases in the midgut is the key factor in exerting their insecticidal activities [23,24]. The difference is that domains IV and V of Cry-1-type proteins are released, while domains I and II of Vip3 proteins are removed after proteolytic processing [23,24,25]. Over-digestion or insufficient processing of Cry protoxins has been reported to affect their virulence [26]. In addition, Vip3Aa mutants with greater stability have been shown to exhibit higher toxicity against *S. frugiperda* and *H. armigera* [27]. In the present study, the addition of EPS delayed proteolytic processing of Cry1Ac and Vip3Aa proteins in the gut juice of *H. armigera* and *S. frugiperda* (Figure 5), which was related to their enhanced toxicity. Because EPS formed specific bonds with Cry1Ac [8] and Vip3A proteins (Figure 4), we speculated that EPS might cover the proteolytic processing sites and make them inaccessible. This is consistent with the extracellular polysaccharide alginate of *Pseudomonas aeruginosa* protecting lipase LipA from degradation by the extracellular protease elastase covering cleavage sites [28].

In our previous study, EPS-HD270 was found to specifically bind to Cry1Ac protoxin (*K*_d_ values of 113.0 ± 35.1 nmol/L) [8] and promote the binding of Cry1Ac to BBMVs of *P. xylostella*. Vip3Aa protoxin showed a higher affinity for EPS (*K*_d_ values of 6.43 ± 1.31 nM) than Cry1Ac (Figure 4), and binding to BBMVs of *H. armigera* and *S. frugiperda* was promoted by interaction with EPS-HD270 (Figure 6). The insecticidal activity of the Vip3Aa protein increased with the promotion of its ability to bind to BBMVs [27].

Domains IV and V of Vip3Aa are predicted to contain a carbohydrate-binding motif [13]. In this study, EPS-HD270 was found to show a higher affinity for Vip3Aa than Cry1Ac (Figure 4). Moreover, it promoted the binding of the Vip3Aa protoxin to BBMVs of *S. frugiperda* and *H. armigera*. (Figure 6). Since the insecticidal activity of the Vip3Aa protein increased with the promotion of its ability to bind to BBMVs [27], EPS-HD270 was found to specifically bind to the Cry1Ac protoxin and promote the binding of Cry1Ac to the BBMV of *P. xylostella* [8]. We speculate that EPS-HD270 increased the virulence of Vip3Aa by enhancing its binding to BBMVs.

## 4. Conclusions

In this study, we found that the extracellular polysaccharide from Bt HD270, a strain belonging to subsp. *kurstaki*, can not only enhance the virulence of insecticidal proteins with different structures commonly contained in subsp. *Kurstaki*, but it also has a synergistic effect against a variety of lepidopteran insect pests. The synergistic activities of EPS-HD270 with Cry-1 type and Vip3Aa proteins were mainly related to the delay in proteolytic processing and promotion of binding to BBMVs.

## 5. Materials and Methods

### 5.1. Strains

Bt serovar *kurstaki* HD270 was used for EPS production. The recombinant Bt strain HD73^−^-*cry1Ab* (Bt HD73-strain containing the *cry1Ab* gene) and the HD73^-^ strain containing the *cry1Ah* gene were used for Cry1Ab and Cry1Ah protein extraction. The Vip3Aa11 protein was derived from an *Escherichia coli* expression system, and the plasmid pET28a carried the *vip3Aa11* gene. All of the strains mentioned here were stored in our laboratory. Luria–Bertani (LB) media were used for all strains to grow at 30 °C.

### 5.2. Isolation and Purification of Insecticidal Proteins

Bt strains HD73-*cry1Ab* and HD73-*cry1Ah* were grown in 1/2 liquid Luria–Bertani (LB) media until 50–60% of insecticidal crystals were released. Cry1Ab and Cry1Ah proteins were extracted according to the continuous crystal solubilization method mentioned by Zhou et al. [29], purified using an ÄKTA avant 150 system (GE Healthcare Life Sciences; Piscataway, NJ, USA) and affinity chromatography (HiTrap Q Sepharose High Performance 5 mL, GE Healthcare, Uppsala, Sweden). Vip3Aa11 protein was extracted and purified according to a previous publication by Wang et al. [30]. Vip3Aa protein carrying a His-tag was purified by Ni^2+^-affinity chromatography and finally desalted. The concentrations of Cry1Ab/h and Vip3Aa11 purified proteins were measured via sodium dodecyl sulfate-polyacrylamide gel electrophoresis (SDS-PAGE) and quantified using ImageJ software (Version 1.42I).

### 5.3. Culture of Strain and Preparation of EPS-HD270

The extraction and purification of EPS-HD270 were performed with reference to the description published by Wang et al. [8]. The Bt serovar *kurstaki* HD270 strain, kept in our laboratory, was cultured at 30 °C using liquid LB medium. The bacterial suspension was collected via centrifugation at 12,000× *g* for 30 min before all spores were released. The supernatant obtained after centrifugation was mixed with 95% ethanol at a volume ratio of 1:3 and placed at 4 °C overnight to precipitate EPS. After centrifugation, the precipitate was resuspended in ultra-pure water, and proteinase K was added at one-tenth of the protein mass in the mixture and digested at 50 °C for 2 h. Proteinase K was then inactivated for 20 min at 100 °C to obtain a crude EPS solution.

The proteinase K-treated solution was purified on an ÄKTA avant 150 system. After small molecule impurities had been removed using a desalting column, the targets were purified using an anion-exchange chromatography column (Q HP column). All fractions were collected and identified via the phenol–sulfuric acid method [31]. The target samples were further separated using a gel filtration chromatography column (HiLoad 26/600 Superdex 200, GE Healthcare Life Sciences). The main components were recovered, and NaCl was removed using a desalting column. EPS-HD270 concentrations were tested using the phenol–sulfuric acid method.

### 5.4. Bioassay

The first instar larvae of *H. armigera* were provided by the Jilin Academy of Agricultural Sciences. The first instar larvae of *S. frugiperda* and the second instar larvae of *P. xylostella* were maintained in our laboratory. Bioassays were performed using proteins, EPS and mixtures of proteins with EPS.

The bioassay method for *P. xylostella* was conducted as mentioned previously [8]. Briefly, fresh cabbage leaves with a diameter of 6 cm were immersed in a gradient of different concentrations of proteins and EPS. The leaf surfaces were dried, and each leaf was placed in a plastic Petri dish (9 cm diameter) with 30 larvae. Cry1A buffer (20 mmol/L Na_2_CO_3_-NaHCO_3_, pH 9.8) and EPS buffer (ultra-pure water) were used as controls. Mortality was calculated 3 days later. Each treatment was performed in three replicates. Thirty larvae were used for each replicate.

The bioassays of *S. frugiperda* and *H. armigera* were performed using first instar larvae [27]. Artificial diets containing soy flour, wheat bran, yeast, and vitamins were prepared as described by Liang et al. [32]. Fifteen grams of artificial diets were weighed separately in each Petri dish (9 cm diameter). The protein–exopolysaccharide mixture (3 mL) was added to the diet and equally distributed into 24-well culture plates after moderate moisture was evaporated. The larvae were placed in the treated diet and covered with tissue. Each treatment was conducted in three replicates and 24 larvae were used for each replicate. The same ratio of Cry1Ac/Vip3Aa11 buffer (20 mmol/L Na_2_CO_3_-NaHCO_3_/20 mmol/L Tris-HCl) and EPS buffer (ultra-pure water) was used as the control. Seven-day mortality was counted.

In all insect bioassay experiments, larvae that did not respond to being poked were determined to be dead larvae.

### 5.5. Preparation of BBMVs

The insects were maintained until the third instar, and the midguts were obtained after dissection. After the preparation of BBMVs via magnesium precipitation [33], the enzyme activity of aminopeptidase N (APN) was calculated to determine the purity of the BBMVs [34].

### 5.6. ELISA Analysis of the Binding of Vip3Aa11 Protoxin to EPS-HD270

EPS-HD270 (2.0 mg/mL, 100 μL) was loaded into 96-well ELISA plates (Nunc Maxisorb, Thermo) and immobilized at 4 °C overnight. The ELISA plates were washed three times with Tris-buffered saline (TBS) and blocked with TBST (TBS containing 0.1% Tween-20) containing 2.0% BSA (200 μL) at 37 °C for 2 h. The plates were washed with TBST three times and incubated with different concentrations of Vip3Aa11 (0, 0.5, 1, 2, 4, 8, 16, 32 nmol/L) (100 μL) for 1 h at 37 °C. After washing with TBST buffer 3 times, anti-Vip3A antibody (TBST 1:5000 dilution, 100 μL) was added. TBST (100 μL) containing 1/10,000 HRP-conjugated goat anti-mouse IgG (Solarbio Life Sciences, Beijing, China) was incubated at 37 °C for 1 h. For each treatment, three replicates were performed. After washing, the reaction was tested with 3,3′,5,5′-tetramethylbenzidine (TMB) solution (Solarbio Life Sciences, Beijing, China) (100 μL) for 15 min in the dark at 37 °C. The reaction was terminated with HCl (2.0 mol/L, 100 μL), and the absorbance was immediately read at 450 nm using a microplate reader. The equilibrium dissociation constant (*K*_d_) was analyzed using Sigma-plot Software (Version 12.0).

### 5.7. Western Blot Analysis of the Binding of Vip3Aa11 Protoxin with EPS-HD270 to BBMVs of S. frugiperda and H. armigera

The EPS-HD270 was incubated with Vip3Aa11 protoxin at 4 °C overnight after mixing with BBMVs (20 µg). The control was EPS buffer (ultrapure water) incubated with BBMVs (20 μg) and Vip3Aa11 protoxin. After centrifugation at 18,000*× g* for 10 min at 4 °C, proteins were separated by SDS-PAGE and electrotransferred to PVDF membranes. After blocking with TBST containing 5% skimmed milk powder on a low-speed spinner for 1 h, the PVDF membranes were incubated with Vip3A antibody at a 1:5000 dilution for 1 h and washed with TBST. HRP-conjugated goat anti-mouse IgG at a 1:10,000 dilution in TBST was added and incubated for 1 h. After they had been washed three times, the membranes were detected with SuperSignal™ West Pico PLUS Chemiluminescent Substrate chemiluminescent substrate (Thermo Scientific, Waltham, MA, USA) using a LAS-4000 mini-imaging system (GE Healthcare, Chicago, IL, USA).

### 5.8. Proteolytic Processing Analysis of Insecticidal Protoxin

The fresh intact midguts of *S. frugiperda* and *H. armigera* third instar larvae were centrifuged at 14,000*× g* for 20 min at 4 °C to obtain the supernatant. The concentration gradient of protein to midgut juice was set at a volume ratio, and the final volume of protein was 100 μL. EPS was added at the mass ratio (EPS: protoxin) used in the bioassay, and the final volume of EPS was 100 μL. The digestion of proteins by the midgut fluid was analyzed via SDS-PAGE after 1 h of incubation in a water bath at 30 °C.

### 5.9. Data Analysis

For each treatment, mortality data of five different concentrations were used to calculate LC_50_ by SPSS 20 (IBM, Armonk, NY, USA) using probit analysis. The statistical analyses in the figure were performed using GraphPad Prism (version 7.0). Before the analysis was performed, percentages were arcsine square root transformed, and pairwise comparisons were performed via *t*-test.

## Figures and Tables

**Table 1:** The LC_50_ value of Cry1Ac and crude EPS against *S. frugiperda* and *H. armigera.*

Insect Pests	Treatment	LC_50_ (μg/g) (95% Fiducial Limits)	Slope ± SE	χ^2^	Fold Change
*S. frugiperda*	Cry1Ac	68.26 (46.95–97.97)	2.67 ± 0.30	7.25	2.03
	Cry1Ac + EPS	33.64 (29.76–37.60)	6.88 ± 0.83	5.31	
*H. armigera*	Cry1Ac	12.40 (9.57–16.49)	1.22 ± 0.17	2.30	2.09
	Cry1Ac + EPS	5.92 (4.28–8.47)	1.72 ± 0.19	3.17	

The concentration of EPS in artificial diets was 2 mg/g. Three replications of each sample.

**Table 2:** The LC_50_ values of Vip3Aa and crude EPS against *S. frugiperda* and *H. armigera.*

Insect Pests	Samples	LC_50_ (μg/g) (95% Fiducial Limits)	Slope ± SE	χ^2^	Fold Change
*S. frugiperda*	Vip3Aa	1.22 (0.96–1.58)	1.34 ± 0.17	1.30	2.49
	Vip3Aa + EPS	0.49 (0.34–0.70)	0.92 ± 0.16	0.74	
*H. armigera*	Vip3Aa	12.57 (9.62–16.98)	1.17 ± 0.17	1.58	2.28
	Vip3Aa + EPS	5.51 (4.28–7.20)	1.25 ± 0.17	2.63	

The concentration of EPS in the artificial diet was 2 mg/g. Three replications of each sample.

## Data Availability

No new sequencing data were created or analyzed in this study.

## Supplementary Materials

The following supporting information can be downloaded at: https://www.mdpi.com/article/10.3390/toxins15100590/s1, Figure S1. The preparation flow diagram of purified EPS-HD270.
