# Supplementary material for: Analysis of Synergism between Extracellular Polysaccharide from Bacillus thuringensis subsp. kurstaki HD270 and Insecticidal Proteins
Source: Toxins (Basel). 2023 Sep 28;15(10):590. doi: 10.3390/toxins15100590 (PMC10610938; doi:10.3390/toxins15100590)
Supplement: Supplementary file 1 [file toxins-15-00590-s001.zip › toxins-2600427-supplementary.pdf]

Analysis of Synergism between Extracellular Polysaccharide from *Bacillus thuringensis* subsp. *kurstaki* HD270 and Insecticidal Proteins.

Bai Xue [1,2], Meiling Wang [2], Zeyu Wang [2], Changlong Shu [2], Lili Geng [2,*] and Jie Zhang [1,2,*]

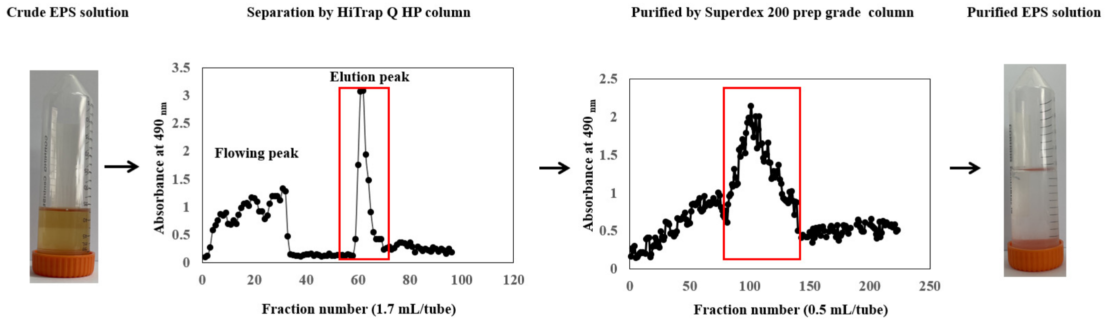

Figure S1. The preparation flow diagram of purified EPS-HD270.
